# Synthesis of 1,8-naphthalimide-based probes with fluorescent switch triggered by flufenamic acid

**DOI:** 10.1098/rsos.172137

**Published:** 2018-06-20

**Authors:** G. Saito, D. Velluto, M. Resmini

**Affiliations:** 1 Department of Chemistry and Biochemistry, SBCS, Queen Mary University of London, E1 4NS London, UK; 2 Diabetes Research Institute and Cell Transplant Center, University of Miami Leonard M. Miller School of Medicine, Miami, FL 33136, USA

**Keywords:** fluorescence, naphthalimide, pH switch, flufenamic acid

## Abstract

In this work, we report the synthesis of novel fluorescent molecules, based on 1,8-naphthalimide thio- and amino-derivatives, designed to display an OFF-ON and ON-OFF photoelectron transfer fluorescence switch upon interaction with a proton-donor drug. We have functionalized the new probes to allow easy formation of a covalent link to polymer matrices, for future applications as drug delivery sensors. We have investigated the fluorescent switch of the new probes using flufenamic acid (FA, pKa 3.65), a water insoluble, non-steroidal anti-inflammatory drug, as a model drug and proton source. The rapid interaction of the new probes with FA resulted in fluorescence enhancement or decrease and a large Stokes shift, all of which allowed the detection of the drug in the nanomolar range. The new 1,8-naphthalimide fluorescent dyes reported in this work are interesting tools for the detection and quantification of acidic drugs and biomolecules.

## 1. Introduction

Over the last two decades, fluorescence has been exploited as a promising method for monitoring chemical species in biological environments, such as fluorescent real-time sensing technologies used in biotechnology and clinical chemistry, especially for DNA sequencing, high-throughput screening and also clinical diagnostics [1–5]. Fluorescence-based techniques are preferred over other methods, because they offer advantages, such as high sensitivity and selectivity, non-invasiveness, real-time monitoring, rapid response and low detection limits [6,7]. One of the use of fluorescence that is common in medical applications is the non-invasive monitoring of clinically relevant species at physiological parameters in the human body [8]. Recently, with the advances in the field of nanotechnology and in particular nanomaterials for biomedical applications, fluorescence has been used for labelling nanoparticles and studying their distribution both *in vitro* and *in vivo* [9].

In this work, we investigated some 1,8-naphtalimide derivatives that have interesting fluorescent properties and can be used as an effective tool for the monitoring and quantification of compounds. Indeed, they have been shown to have high sensitivity to solvent effects and a good ability to bind to substrates [10,11]. Naphthalimides, due to their unique properties, have found applications in many areas of chemistry, such as fluorescent sensors, dyes, chemical probes, sensing of biologically relevant cations and anions, and also in the pharmaceutical field as anti-cancer treatments [1,2,12].

The optical and photophysical properties of 1,8-naphthalimides are very sensitive to any changes in the chemical structure of the aromatic ring via the addition of substituents [12,13]. In particular, the introduction of amines to the 1,8-naphthalic-anhydride core has been extensively explored because the proton input results in alteration of fluorescence properties [14,15]. In addition to chemical modifications, the emission spectra of many fluorophores are sensitive to the polarity of the surrounding environment (solvent effects) [8,11,14]. This phenomenon also affects their ability to switch fluorescence and the overall magnitude of fluorescence enhancement or quenching that can be observed [16].

One of the most commonly exploited approaches for the design of novel fluorescent tags is based on the photoinduced electron transfer process, also known as PET. In this system, the absorption of light leads the excited electrons to jump from the receptor to another molecule or component of the composite system, generating the effect; when an architecture with a short spacer is used, reasonably fast PET rates can be obtained [17–19]. Chemical modifications to incorporate different functional groups can be readily carried out on either the aromatic naphthalene moiety itself or at the *N*-imide site, to tailor the tags to the different applications [20,21]. Depending on the chemical groups linked to the naphthalimide core, significant changes in the photophysical properties can be recorded, resulting in the reversible OFF-ON or ON-OFF transition of the switches upon a chemical input or a compound. When the receptor is unbound, the molecule dims or enhances its fluorescence due to PET from the receptor to the fluorophore [18,22]. Currently, there is a strong interest in expanding the range of synthetic methodologies that allow fast conjugation of dyes and/or fluorescent tags to nanocarriers for applications in drug delivery [23]. Delivery systems that contain both an active drug and a fluorophore based on 1,8-naphthalimides have been developed, as well as different nanoparticles with covalently immobilized analyte-sensitive fluorophores [9,24]. However, the full potential of these fluorescent tags has yet to be fulfilled.

**Figure 1.**
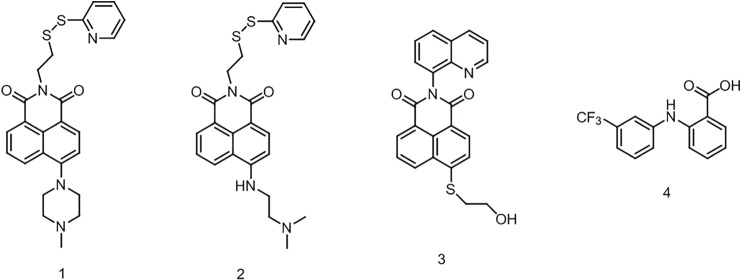
Chemical structures of OFF-ON probes (1 and 2), ON-OFF probe (3) and FA (4).

In this work, we report the design and development of three naphtalimide probes based on either the ON-OFF or the OFF-ON PET switching, by exploiting the functionalization of the main core with amino- and quinoline derivatives as proton receptors as well as the addition of linker groups designed to allow conjugation to polymers or macromolecular assemblies, for potential drug sensing [25,26]. Demonstration of the potential for these new probes to be used for the monitoring of drug loading/release was achieved using flufenamic acid (FA), a water insoluble non-steroidal anti-inflammatory drug, as model (figure 1).

## 2. Material and methods

4-Bromo-1,8-naphthalic anhydride, FA, ethanethiol, cysteamine hydrochloride, 2-mercaptoethanol, aldrithiol-2, 1-methylpiperazine, *N*,*N*-dimethylethylendiamine, 8-aminoquinoline, 2-hydroxyethyl disulfide, tris(2-carboxyethyl)phospine hydrochloride, potassium hydroxyde and triethylamine were purchased from Sigma-Aldrich (UK); ethanol, methanol, dichloromethane ethyl, acetate, acetone, acetonitrile and the low iron silica sand were purchased from Fluka (UK). Diethyl ether, *n*-hexane, dimethylformamide, tetrahydrofurane (HPLC grade) and the silica gel were purchased by VWR. Potassium carbonate was provided by Alfa Aeser (UK). Ultra-dry dimethyl sulfoxyde 99.7% and sodium hydroxide were obtained by Fisher scientific (UK). 2-Methoxyethanol from Honeywell Riedel-de Haen (UK). Chloroform-D and dimethyl sulfoxide-D6 by Cambridge Laboratories (UK). All the reagents were used without further purification.

### 2.1. Synthesis of OFF-ON fluorescent probes

The first part of this work focused on the synthesis of two types of naphthalimide-based OFF-ON switchable probe (1 and 2), designed according to the fluorescence enhancement principle [18].

#### 2.1.1. Preparation of probe 1

##### 2.1.1.1. Synthesis of 2-(pyridin-2-yldisulfanyl) ethanaminium chloride (compound 5)

**Scheme 1.**
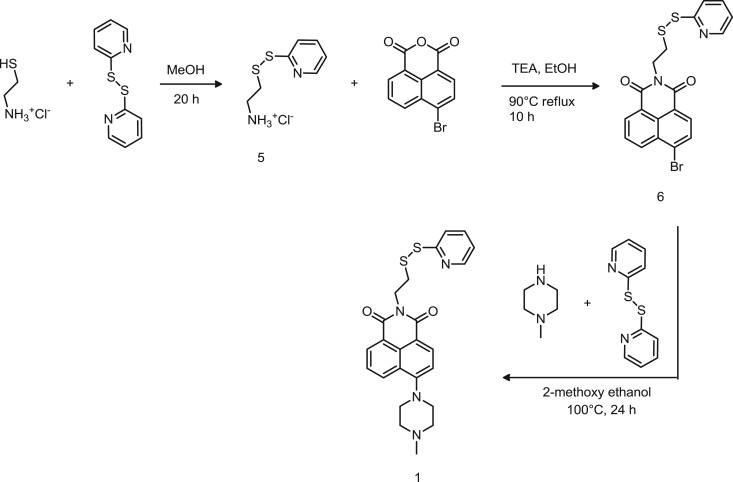
Synthetic route to probe 1.

Cysteamine HCl (0.57 g–0.5 mmol) was dissolved in 10 ml of MeOH in the presence of an excess of aldrithiol-2 (3.30 g–15 mmol). The yellow solution was stirred overnight and the reaction monitored by TLC. The final product was purified by double precipitation in 500 ml of Et_2_O. The precipitate was filtered on filter paper and was dried under reduced pressure. A white powder was obtained (0.94 g) with a chemical yield of 84.0%. ^1^H-NMR (400 MHz, DMSO-d_6_): *δ* 8.52 (1H, dd, *J* = 1.2, 4.8), 8.13 (3H, s), 7.85 (1H, m), 7.75 (1H, d, *J* = 8.0), 7.30 (1H, m), 3.32 (2H, s), 3.09 (2H, s). The reaction is described in scheme 1.

##### 2.1.1.2. Synthesis of 6-bromo-2-(2-(pyridin-2-yldisulfanyl)ethyl)-1H-benzo[de]isoquinoline-1,3(2H)-dione (compound 6)

4-Bromo-1,8-naphthalic anhydride (3.91 g–14.14 mmol) was suspended in ethanol (210 ml) and pyridin-disulfanyl ethanamine (5) (3.15 g–14.14 mmol) was also introduced in the reaction flask. An excess of triethylamine (2.25 ml–15.8 mmol) was added to deprotonate the cysteamine and form the amide with the anhydride. The reaction was allowed to stir for 10 h at 90°C under reflux while monitoring the formation of the product by TLC in ethyl acetate/hexane 30 : 70 mixture. Once completed, the suspension was then filtered, washed with ethanol and then dried under vacuum. A pale brown powder was obtained (4.05 g) with a chemical yield of 64.3% and immediately used for the next step.

^1^H-NMR (400 MHz, CDCl_3_): *δ* 8.60 (1H, d, *J* = 7.2), 8.52 (1H, d, *J* = 8), 8.43 (1H, d, *J* = 4), 8.34 (1H, d, *J* = 8), 8.00 (1H, d, *J* = 8), 7.81 (1H, t, *J* = 7.2, 8), 7.67 (1H, d, *J* = 8), 7.57 (1H, t, *J* = 7.6, 7.6), 7.04 (1H, t, *J* = 6.0, 5.8), 4.52 (2H, t, *J* = 6.8, 7.2), 3.15 (2H, t, *J* = 7.2, 6.8). ^13^C NMR (100 MHz, CDCl_3_) *δ* 163.46 (s, 2C), 159.83 (s), 149.76 (s), 136.90 (s), 133.45 (s), 132.22 (s), 131.38 (s), 131.13 (s), 130.61 (s), 130.52 (s), 128.97 (s), 128.11 (s), 122.81 (s), 121.93 (s), 120.73 (s), 120.04 (s), 39.43 (s), 36.21 (s). FT-IR: cm-1 1699.1 (─C═N), 1656.3 (–N─C═O), 1569.3 (─C═C─Ar), 1339.1 (─C─N), 780.2 (─C─Br).

##### 2.1.1.3. Synthesis of 6-(4-methylpiperazin-1-yl)-2-(2-(pyridin-2-yldisulfanyl)ethyl)-1H-benzo[de]isoquinoline- 1,3(2H)-dione (compound 1)

Product 6 (4.046 g–9.078 mmol) was dissolved in 2-methoxy ethanol (180 ml). An excess of 1-methylpiperazine (15 ml–136 mmol) and aldrithiol-2 (20 g–90.78 mmol) was added and the solution was refluxed at 100°C for 24 h. A dark brown solution was formed, cooled down via the addition of ice cold water (1.8 l). The solution was extracted using dichloromethane and concentrated under reduced pressure. The crude product was purified by gel chromatography first using acetone/dichloromethane 40 : 60, followed by methanol/dichloromethane 5 : 95 to yield two pure yellow products that were collected separately as two yellow powders, after removal of the solvent by evaporation under reduced pressure. Product A (compound 1) of 1.64 g, chemical yield 39.3%. Product B (dimer, electronic supplementary material, figure S3) of 1.01 g, chemical yield 31.2%.

Product A: 6-(4-methylpiperazin-1-yl)-2-(2-(pyridin-2-yldisulfanyl)ethyl)-1H-benzo[de]isoquinoline-1,3(2H)-dione

^1^H-NMR (400 MHz, CDCl_3_): *δ* 8.50 (1H, dd, *J* = 0.8, 7.4), 8.43 (1H, d, *J* = 8), 8.41 (1H, dd, *J* = 0.8, 4.8), 8.36 (1H, dd, *J* = 0.8, 8.4), 7.64 (2H, m), 7.54 (1H, ddd, *J* = 1.6, 7.6, 7.6), 7.16 (1H, d, *J* = 8.0), 7.01 (1H, ddd, *J* = 0.8, 7.6, 8), 4.49 (2H, t, *J* = 7.2), 3.27 (4H, s), 3.15 (2H, t, *J* = 7.2), 2.71 (4H, s), 2.40 (3H, s). ^13^C NMR (100 MHz, CDCl_3_) *δ* 164.39 (s), 163.86 (s), 160.06 (s), 156.18 (s), 149.71 (s), 136.89 (s), 132.82 (s), 131.30 (s), 130.54 (s), 129.96 (s), 126.16 (s), 125.64 (s), 122.99 (s), 120.63 (s), 119.95 (s), 116.33 (s), 115.00 (s), 55.14 (s, 2C), 53.00 (s, 2C), 46.15 (s), 39.21 (s), 36.40 (s). FT-IR: cm^−1^ 2922.6 (─C─H Al), 1693.1 (─C═N─), 1653.2 (–N─C═O), 1573.7 (─C═C─Ar), 1137.8 (C─N), 693 (─CH_2_–S). Melting point range 156–165°C.

#### 2.1.2. Synthesis of probe 2

##### 2.1.2.1. Synthesis of 6-((2-(dimethylamino)ethyl)amino)-2-(2-(pyridin-2-yldisulfanyl)ethyl)-1H-benzo[de]isoquinoline- 1,3(2H)-dione (compound 2)

Product 6 (0.50 g–1.12 mmol) was suspended in 2-methoxyethanol (10 ml) in the presence of *N*′, *N*′-dimethylethylendiamine (1.226 ml–11.22 mmol) and aldrithiol-2 (2.472 g–11.22 mmol). The suspension was stirred for 4 h at 80°C until it turned into a dark red-brown solution. The solution was cooled to room temperature and the solvent was removed by evaporation under reduced pressure to obtain the crude product. The pure compound was obtained by gel chromatography using dichloromethane/ethanol 96 : 4 as mobile phase and the pure solid product was obtained by evaporation of the solvent under reduced pressure. The chemical yield was 8.2% (0.042 g).

^1^H-NMR (400 MHz, CDCl_3_): *δ* 8.52 (1H, dd, *J* = 1, 8), 8.43 (1H, dd, *J* = 1, 4.8), 8.39 (1H, d, *J* = 8.4), 8.24 (1H, dd, *J* = 1, 8), 7.70 (1H, dd, *J* = 1, 8), 7.61 (1H, dd, *J* = 1, 8), 7.54 (1H, dd, *J* = 1, 8), 7.03 (1H, ddd, *J* = 1, 4.8, 8), 6.62 (1H, d, *J* = 8.4), 6.52 (1H, t, *J* = 4.4), 4.50 (2H, t, *J* = 7.2), 3.43 (2H, q), 3.17 (2H, t, *J* = 7.2), 2.83 (2H, t, *J* = 6), 2.42 (6H, s). ^13^C NMR (100 MHz, CDCl_3_) *δ* 164.78 (s), 164.11 (s), 160.41 (s), 150.01 (s), 149.90 (s), 137.01 (s), 134.94 (s), 131.47 (s), 130.07 (s), 126.80 (s), 124.81 (s), 122.93 (s), 120.72 (s), 120.66 (s), 120.04 (s), 109.91 (s), 104.61 (s), 57.02 (s), 45.16 (s, 2C), 40.26 (s), 39.28 (s), 36.71 (s). Melting point range 110–114°C.

### 2.2. Synthesis of ON-OFF fluorescent probe

The second part of this work focused on the preparation of one naphthalimide-based ON-OFF switchable probe.

#### 2.2.1. Synthesis of probe 3

##### 2.2.1.1. Synthesis of 6-bromo-2-(quinolin-8-yl)-1H-benzo[de]isoquinoline-1,3(2H)-dione (compound 7)

4-Bromo-1,8-naphthalic anhydride (1.0 g–3.60 mmol) and 8-aminoquinoline (0.52 g–3.60 mmol) were suspended in methanol (37.5 ml) and reacted by heating under reflux for 24 h. TLC monitoring in ethyl acetate/hexane 30 : 70 confirmed that the starting material was consumed. The reaction was cooled down and the precipitate was filtered and washed first with hexane and then with acetonitrile; 0.385 g of pure product 7 (grey powder) was obtained with a chemical yield of 38.57%. ^1^H-NMR (400 MHz, CDCl_3_): *δ* 8.80 (1 H, dd, *J* = 1.6, 4.4), 8.72 (1 H dd, *J* = 1.2, 7.8), 8.66 (1 H, dd, *J* = 0.8, 8.2), 8.48 (1 H, d, *J* = 7.6), 8.25 (1 H, dd, *J* = 0.8, 8.4), 8.10 (1 H, d, *J* = 7.6), 7.99 (1 H, dd, *J* = 1.6, 8.0), 7.90 (1 H, dd, *J* = 7.2, 8.4), 7.75 (2H, m), 7.42 (1 H, dd, *J* = 4.0 ,8.4). ^13^C NMR (100 MHz, CDCl_3_) *δ* 164.36 (s, 2C), 151.03 (s), 144.20 (s), 136.29 (s), 133.55 (s), 132.45 (s), 131.61 (s), 131.16 (s), 130.96 (s), 130.56 (s), 130.00 (s), 129.90 (s), 129.40 (s), 129.31 (s), 128.14 (s), 126.31 (s), 123.58 (s), 122.70 (s), 121.79 (s).

##### 2.2.1.2. Synthesis of 6-((2-hydroxyethyl)thio)-2-(quinolin-8-yl)-1H-benzo[de]isoquinoline-1,3(2H)-dione (compound 3)

*N*-(Quinolin-8-yl)-4-bromo-1,8-naphthalimide (7) (0.20 g–0.50 mmol) was dissolved in dimethylformamide (15 ml) in the presence of potassium carbonate anhydrous (0.10 mg–0.75 mmol). An excess of mercaptoethanol (52.6 µl–0.75 mmol) was added to the mixture under nitrogen atmosphere and was stirred at 40°C for 24 h. The product, 0.12 g of a yellow powder, was recovered by precipitation in water and filtration followed by vacuum drying. The chemical yield was 60.81%. ^1^H-NMR (400 MHz, CDCl_3_): *δ* 8.80 (1H, dd, *J* = 1.6, 4.2), 8.70 (2H, m), 8.54 (1H, d, *J* = 7.6), 8.24 (1H, dd, *J* = 1.6, 8.4), 7.98 (1H, dd, *J* = 1.6, 8.0), 7.82 (1H, dd, *J* = 7.2, 8.4), 7.78 (1H, dd, *J* = 1.6, 7.2), 7.71 (2H, m), 7.42 (1H, dd, *J* = 4.0, 8.0), 3.96 (2H, dd, *J* = 6.4, 6.0), 3.40 (1H, t, *J* = 6.0), 2.04 (1H, t, *J* = 6.0). ^13^C NMR (100 MHz, CDCl_3_) *δ* 164.53 (s), 164.49 (s), 164.30 (s), 151.15 (s), 144.40 (s), 144.01 (s), 136.44 (s), 133.84 (m), 132.19 (s), 131.21 (s), 130.67 (s), 130.53 (s), 130.19 (s), 129.51 (s), 129.47 (s), 129.44 (s), 127.09 (s), 126.47 (s), 124.40 (s), 123.81 (s), 121.88 (s), 60.57 (s), 35.79 (s). FT-IR: cm^−1^ 3398.5 (-OH), 1696.6 (─C═N─), 1639.3 (–N─C═O), 1582.6 (─CH=CHAr), 1366.0 (─CH), 1240.4 (─C─N), 1054.5 (─C─OH).

## 3. Results and discussion

### 3.1. Probes design and synthesis

Probes 1 and 2 were designed based on a fluorophore–spacer–receptor architecture, in which the electron transfer from the receptor to the fluorophore controls the photophysical switch of the fluorophore [17–19]. For the OFF-ON switch, an electron-releasing group has been integrated at position 4 of the naphthalimide ring (scheme 1), such as to turn ON the fluorescence emission upon a proton input [27]. The fluorescence is set back to OFF when the input is removed.

In particular, we investigated the substitution of the 1,8-naphthalimides with either a piperazine residue (probe 1) or with an ethylene diamine (probe 2) in position 4.

In both probes 1 and 2, the naphthalimide was obtained by reacting the anhydride with a cysteamine protected with a 2,2'-dipyridyldisulfide (PDS), to form the cysteamine pyridyldisulfide (compound 5, scheme 1). The formation of the cysteamine-PDS was obtained by a disulfide exchange and was proven by the disappearance of the free thiol signal *δ* 1.50 ppm and the shift of aliphatic protons of the cysteamine at *δ* 3.09 and 3.32 ppm in the ^1^H-NMR spectrum (electronic supplementary material, figure S1). The naphthalic anhydride was then reacted with the cysteamine-PDS in basic conditions at 90°C to favour the nucleophilic substitution, and the reaction was monitored by TLC to confirm that the starting materials were consumed after 10 h. ^1^H-NMR of the product showed the shifts of the two triplets (─CH2 at *δ* 3.20 and 4.55 ppm) of the ethanaminium of the cysteamine (electronic supplementary material, figure S2) confirming the product formation additionally supported by the small shift of the pyridine protons on the carbon in meta position (C3). Probe 1 was obtained by reacting product 6 and the methylpiperazine in 2-methoxyethanol, selected because is a good solvent for both compounds [28]. After incubating the reaction for 24 h, TLC analysis (ethyl acetate/diethyl ether 40 : 60) showed a yellow fluorescent compound with a retention factor (Rf) of 0.02. However, by using a more polar eluent (methanol/dichloromethane 5 : 95) two distinct fluorescent compounds, with Rf 0.05 and 0.12, respectively, were evidenced by TLC and separated by gel chromatography. Both products were purified by precipitation in water and extraction in dichloromethane. ^1^H-NMR analysis confirmed the formation of product 1 (Rf 0.05) through the shift of the piperazine protons at *δ* 3.27 and 2.71 ppm (electronic supplementary material, figure S2). Dimerization of product 1 due to the release of pyridil sulfone, a stable leaving group formed after displacement by the free thiol terminated probe generated during the synthetic process, resulted in the secondary product shown in electronic supplementary material, figure S3 (Rf 0.12). ^1^H-NMR and LC-MS, where a peak at 708 *m/z* was identified (electronic supplementary material, figure S3), confirmed the formation of the dimer. Attempts to suppress formation of the dimer were done by increasing the temperature (140°C) without success, however the dimer was easily converted into a thiol terminated reagent by reduction of the disulfide with tris(2-carboxyethyl)phosphine hydrochloride 10 mM. Further reaction with an excess of 2,2′-dipyridyldisulfide yielded the desired product 1.

**Scheme 2.**
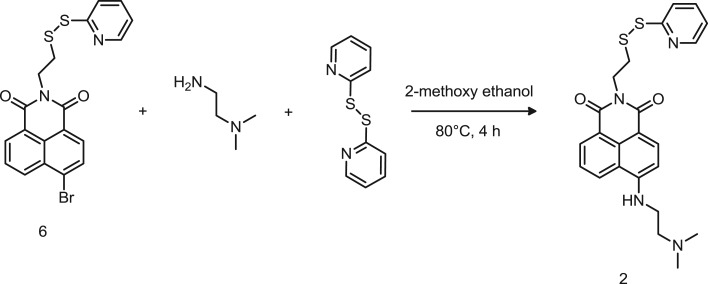
Synthetic route to probe 2.

In order to control the fluorescence switch capability and intensity, we have evaluated a different receptor molecule and we designed probe 2. In this case, an ethylene diamine group was introduced in position 4 of the 1,8-naphthalimide ring, instead of the methylpiperazine residue (scheme 2). Specifically, compound 6 was reacted with an excess of *N*′,*N*'-dimethylethane-1,2-diamine via nucleophilic substitution of the bromine. The resulting solution was purified by gel chromatography using dichloromethane/ethanol 96:4 as mobile phase. The presence of a quartet at *δ* 3.43 and a triplet at 3.17 ppm in the ^1^H-NMR spectrum (electronic supplementary material, figure S4) confirmed the formation of compound 2 although with a very low chemical yield (less than 10%). As previously observed for product 1, also in this case the formation of the dimer, identified by the LC-MS peak at 685 *m/z* (electronic supplementary material, figure S5), significantly reduced the chemical yields of the desired product. This could be attributed either to the short duration of the reaction or to the low separation between the target molecule and its dimer during the chromatographic purification. No attempts to suppress the dimerization reaction were carried out in this case. Nevertheless, as this synthesis was carried out on a small scale, increase in chemical yields are likely to be significantly improved by applying a combination of the following: working on a larger scale, increasing the reaction time from 4 to 24 h or even longer, and also by maintaining the temperature of the reaction solution at 120°C.

**Scheme 3.**
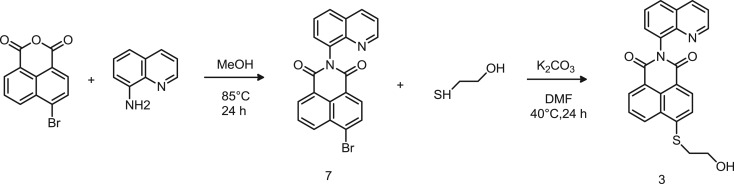
Synthetic route to probe 3.

Probe 3 was designed to undergo quenching upon interaction with protons. 8-Aminoquinoline was reacted with the naphthalic anhydride to yield product 7 (scheme 3), which did not display fluorescence due to the strong electron-withdrawing effect of the bromine in position 4 of the naphthalic ring. The formation of the product was confirmed by the disappearance of the primary amine protons of 8-aminoquinoline. As already reported, the substitution of the bromine with a thiol containing molecule (2-mercaptoethanol) enhanced the fluorescence emission, due to the electron-releasing effect and provided the probe with a free end for further conjugation with biomolecules or polymers [12]. The aliphatic protons of the receptor molecule were identified by the peaks at *δ* 3.96 and the triplet at 3.40 ppm in the ^1^H-NMR spectrum, confirming the formation of probe 3 (electronic supplementary material, figure S6) with a chemical yield of 61%.

### 3.2. Fluorescent probes: characterization and interaction with flufenamic acid as model drug

**Figure 2.**
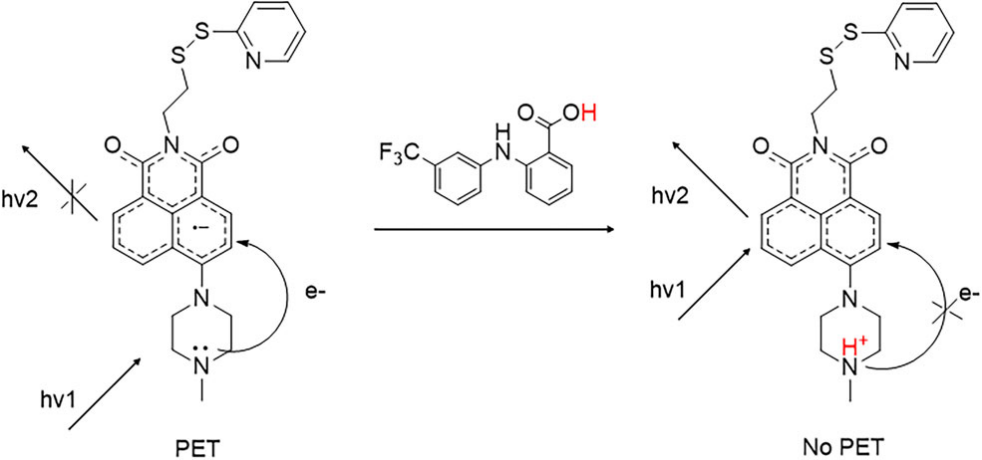
Proposed mechanism for fluorescence enhancement (probes 1 and 2).

Products 1 and 2 were designed to lead to fluorescence enhancement while product 3 to fluorescence quenching. In probes 1 and 2, the protonation of the distal amino group of the 1-methylpiperazine and the *N*′,*N*'-dimethylethylene diamine, respectively, occurs because of the pKa values of the receptors (9.14 ± 0.03 for –N─CH_3_ 1-methylpiperazine and 9.70 for (CH_3_)_2_N─) and it interferes with the PET mechanism causing fluorescence emission enhancement [29,30]. In probe 3, the protonation of the quinoline residue results in the formation of a hydrogen bond between the quinoline and the carbonyl group of the naphthalic core, which interferes with the emission of photons resulting in the observed fluorescence quenching for product 3 [22,31,32]. The proposed mechanism for fluorescence enhancement (probes 1 and 2) is shown in figure 2, while the fluorescence quenching (probe 3) is shown in electronic supplementary material, figure S7.

**Figure 3.**
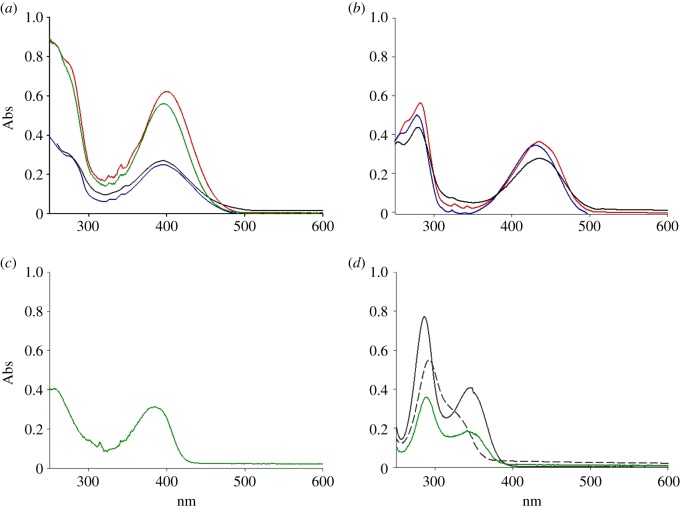
UV–vis traces of probe 1 (*a*) in acetonitrile (53 nM, red line), acetonitrile–phosphate buffer 1 mM pH 7.2 1 : 1 (black line), acetonitrile–H_2_O 1 : 1 (blue line) and tetrahydrofuran (43 nM, green line); probe 2 (*b*) in acetonitrile (31 nM, red line), acetonitrile–phosphate buffer 1 mM pH 7.2 1  : 1 (black line) and acetonitrile–H_2_O 1 : 1 (blue line); probe 3 (*c*) in tetrahydrofuran (11 nM, green line); UV–vis spectra of FA (*d*) in tetrahydrofuran (71 nM, green line), in H_2_O pH 1 (35 µM, grey line) and pH 14 (23 µM, grey dashed line).

In order to evaluate the effect of the solvent polarity on the excitation and emission of the probes, all the probes were dissolved in different solvents such as tetrahydrofuran, acetonitrile, or in combinations such as acetonitrile/water at different ratios, and acetonitrile/1.0 mM phosphate buffer saline at pH 7.2. We observed that the polarity of the environment impacted the maximum excitation wavelength and the intensity of the corresponding fluorescence emission. We found that maximum excitation wavelength ranged between 390 and 398 nm for probe 1 (figure 3*a*), between 428 and 435 nm for probe 2 (figure 3*b*) and around 380 nm for probe 3 (figure 3*c*). According to these observations, the increased solvent polarity led to a characteristic hypsochromic (or blue) shift for probes 1 and 2, so-called negative solvatochromic effect. This may be explained by intermolecular solute/solvent interactions, as a better stabilization by solvation of the ground-state molecule compared to the first excited state of the dyes, resulted in overall increase in the energy gap between the two states [33]. Probes 1 and 2 followed the same trend, with shorter excitation wavelength for molecules dissolved in acetonitrile–H_2_O mixtures than acetonitrile. Unexpectedly, the spectrum of probe 1 in tetrahydrofuran (relative polarity 0.207) resulted in a stronger hypsochromic shift compared with acetonitrile (relative polarity 0.460), which is not in accordance with the solvent polarity scale. No evaluation can be reported for probe 3 due to poor solubility in water-based mixtures.

**Figure 4.**
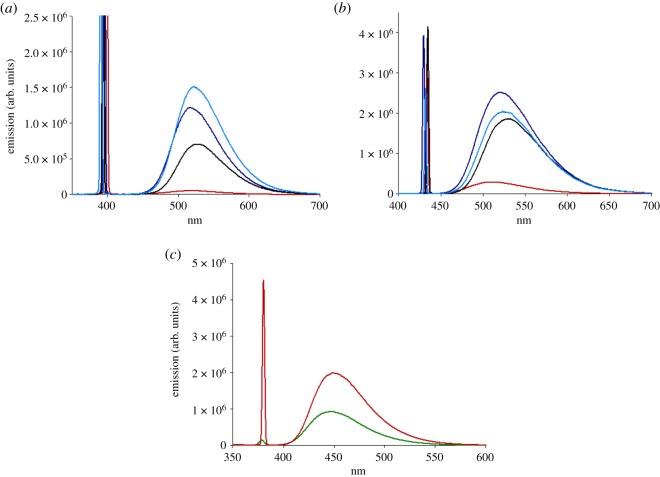
Fluorescent emission of probe 1 (10.7 nM) (*a*), probe 2 (11.0 nM) (*b*) and probe 3 (12.5 nM) (*c*) in acetonitrile (red line), acetonitrile–phosphate buffer 1 mM pH 7.2 1 : 1 (black line), acetonitrile–H_2_O 1 : 1 (dark blue line), acetonitrile–H_2_O 8 : 2 (blue line) and tetrahydrofuran (2.5 nM, green line).

All three dyes analysed by the in-solution study displayed a sensible Stokes' shift. Dyes 1 and 2 exhibited strong green fluorescence emission with a maximum around 520 nm (figure 4*a*,*b*), while probe 3 emitted maximum fluorescent blue light at 450 nm (figure 4*c*). Fluorescence data obtained using the maximum excitation wavelength in solvents with increasing polarity exhibited a bathochromic shift using the same probe concentration (5 µg ml^−1^). In all cases, the emission wavelength shifted towards red light by increasing the water content in the co-solvent mixture, which is in line with reported data [14]. The biggest red shift was observed in acetonitrile–phosphate buffer 1 mM pH 7.2 1 : 1, from 515 nm in acetonitrile to 530 nm for probe 1 and from 513 nm in acetonitrile to 532 nm for probe 2. A very small red shift (up to 3 nm) seems to be displayed when the mixture acetonitrile–H_2_O 8 : 2 is used, compared to the more polar acetonitrile–H_2_O 1 : 1, however, the difference is very small and it might be the result of small local fluctuation in intensity, so it was considered negligible.

Figure 4 also shows the effect of solvent polarity on the maximum emission. An increased water content, by mixing acetonitrile with water in different ratios, resulted in an increased magnitude of fluorescent emission at the same probe concentration. Moreover, dyes 1 and 2 fluoresce more in mixture acetonitrile–H_2_O 1 : 1 than in acetonitrile–phosphate buffer, suggesting a role of the buffer in the stabilization of the system. However a buffered medium with higher ionic strength might impact differently on the fluorescence outcome. Furthermore, the presence of the piperazine group in probe 1 greatly affected the maximum emission compared to the linear amine group of probe 2. The same trend was observed for probe 3, but due to poor solubility in the water-based mixture, a comparison could only be done in the organic solvent.

As the aim of this work was to develop new naphtalimide-based fluorescent tags that could be easily coupled to nanocarriers, potentially for drug delivery applications, the next step focused on the characterization of the interactions using a model drug. The proton releasing FA was chosen, a hydrophobic anti-inflammatory drug, although these probes were not designed to interact specifically with this molecule. The expectation is that these findings will be applicable to any small drug molecule containing an acidic group.

In order to ensure that interference did not occur between the excitation bands of the probes and the FA, the UV–vis spectrum of FA was recorded in different solvent systems such as tetrahydrofuran, acidic and basic aqueous solutions at pH 1 and 14, respectively. The data are shown in figure 3*d* and in all cases no absorption band was observed above 350 nm.

**Figure 5.**
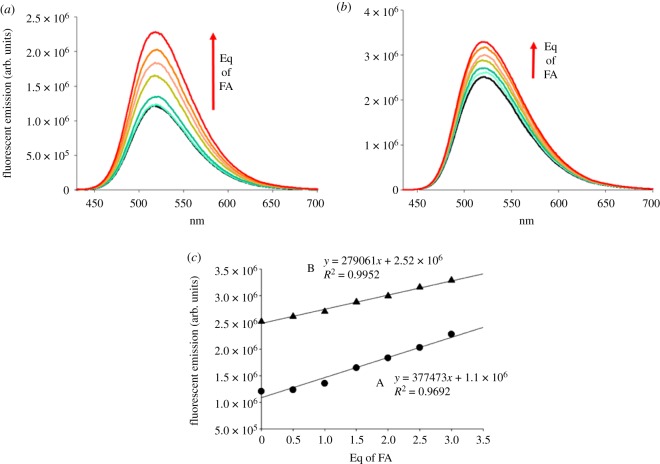
Fluorescent emission spectra of probe 1 = 10.7 nM (*a*) and probe 2 = 11.0 nM (*b*) upon interaction with FA in a 1 : 1 mixture of acetonitrile and water. A linear correlation between the maximum fluorescent emission intensity at 520 nm and the equivalents of FA was obtained in both cases (*c*), indicating a direct dependence of the fluorescence enhancement on the proton input.

The effect of proton input on the fluorescence emission of the three probes was then investigated. A range of FA concentrations between 0.1 and 5 equivalents (molar ratio) compared to the probes were evaluated (figures 4 and 5). Results showed that when the probes are dissolved in tetrahydrofuran at concentration of 5.0 µg ml^−1^ (probe 1 = 10.7 nM and probe 2 = 11.0 nM), even upon addition of FA no fluorescence enhancement is recorded. On increasing the probe concentration by 10-fold the same trend was observed. Presumably the low polarity of the tetrahydrofuran did not allow good excitation in the π system of naphthalimide. A slight emission enhancement of 1.27 times was registered in acetonitrile upon addition of three equivalents of FA to a concentration of probe of 10.7 nM. Although the probe dissolved in the acetonitrile/phosphate buffer mixture showed higher initial fluorescence than in pure acetonitrile, it did not display a consistent increase of fluorescence (1.14-fold) when FA was added. In the buffered medium three equivalents of FA did not cause fluorescent switch, but in a 1 : 1 mixture of acetonitrile/water, the fluorescent intensity was enhanced by 2.04 times for 1 (figure 5). Probe 2 displayed the same behaviour in all the conditions tested, because of the chemical and pKa similarities of the distal amine group with probe 1. Higher fluorescence emission compared to probe 1 was measured in acetonitrile (1.51-fold) without the addition of FA. Probably the electron transfer from the tertiary amine to naphthalic core of probe 2 was favoured by the higher pKa value compared with the receptor in probe 1 and the higher degree of flexibility of the linker, resulting in a higher emission intensity in both organic and mixed organic/water phases [29,30]. In the acetonitrile/H_2_O 1 : 1 mixture, a smaller enhancement (1.32-fold) was observed when compared with probe 1. This was possibly related to greater obstruction of PET when a methylpiperazine residue was bound, as the higher rigidity of a closed six-term ring could reduce the rate of PET transfer. Taken together, these results are in line with the higher fluorescence exhibited in organic solvent for probes with similar chemical structure [16]. This is attributed to the quantum yield falling as the solvent polarity increases. Overall, water plays a role not only in the polarity of the environment, but also it might be responsible for protonation of distal amines, which could result in a higher intensity of emission. Moreover, the fluorescence switching after the protonation of distal amines could be lower than expected as partial protonation might have already occurred [16].

Figure 5*c* shows the direct correlation between the fluorescence enhancement and the equivalents of quencher (FA) that were added for both probe 1 and probe 2. Both probes have low solubility in the system evaluated and attempts to estimate binding and quenching constants were not successful. Plots of F/F_0_ versus [FA] (electronic supplementary material, figure S8, ESI) show that the data acquired are still in the linear portion of the saturation curve. The use of the Benesi–Hildebrand equation to calculate association constants [34] with the available data could not be implemented, as the errors associated with the analysis were too large.

The fluorescence switch was also evaluated for probe 3. Upon a proton input (FA in this work) the quinolinyl residue of the probe becomes a quinolinium cation and, according to the proposed mechanism, the NH^+^ forms a hydrogen bond with the oxygen atom of the carbonyl on the amide inducing fluorescence quenching of the 4-ethanthiol-1,8-naphthalimide derivative. This hypothesis was confirmed by others in a recent study, as it might be easier for the nitrogen atom to form a stable intramolecular hydrogen bond, which bites back into the naphthalimide system and thus leads to efficient fluorescence quenching [22,31,32]. However, no further spectroscopic investigation was carried out to support this hypothesis, so the authors refer to previously published work where the quenching mechanism was reported (electronic supplementary material, figure S7).

**Figure 6.**
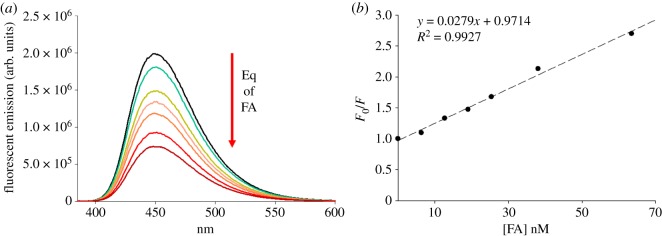
Quenching of probe 3 at concentration 12.7 nM upon interaction with FA in acetonitrile (*a*). Stern–Volmer plot which shows linear correlation (*b*).

The UV–vis spectrum of probe 3 in THF resulted in maximum absorption at a wavelength of *λ* = 380 nm and maximum fluorescence emission in the blue region at 450 nm. The effect of the interaction with the proton source (FA) was evaluated on probe 3 at a concentration of 12.7 nM, having previously proven that a self-quenching effect does not occur at such a low concentration of the probe (data not shown). Addition of FA in a molar ratio up to five equivalents with respect to the fluorophore resulted in the observation of a significant decrease of fluorescence emission as shown in figure 6, with the addition of three equivalents of drug resulting in halving of the fluorescence. The fluorescence quenching data were analysed using the Stern–Volmer equation and plotted as *F*/*F*_0_ [FA] shown in figure 6*b*. The data can be fitted with a linear equation, with a good fit, where the slope provides the value for the Stern–Volmer constant (K_S_) of 2.79 × 10^−7^ M^−1^. The quenching occurring between probe 3 and FA is presumed to occur via static quenching, due to the linearity of the data, and also to the very small size of K_S_ and the fact that the measurement is done at very low concentrations [35]. Under these assumptions, the Stern–Volmer constant can then be considered as the association constant. Lifetime measurements, which would allow a more precise calculation, could not be obtained, however, the literature data for naphtalimide probes [36] report values for the lifetime (*τ*) of these compounds comprised between 10 and 30 ns. If an indicative value of 20 ns is used and applied in conjunction with the Ks, it would result in an estimated value for the quenching constant for probe 3 of 13.95 M^−1^ s^−1^.

## 4. Conclusion

Three fluorescent probes have been designed to integrate a molecular system that allows the PET by including amines as receptors. The probes were successfully synthesized and characterized for their fluorescence properties. We have demonstrated that the probes can be excited and their emission can be turned either ON or OFF upon interaction with a proton source such as a mild acid. Enhancement of fluorescent emission under progressive addition of weak acid (FA pKa = 3.69) was shown for products 1 and 2, as the PET mechanism was interrupted by the protonation of the receptor. The intensity of the switch is different in relation to the pKa of the receptor and the polarity of the environment as fluorescent emission rose 2.04 times for 1 and 1.32 times for 2 in the mixture of acetonitrile/water 1 : 1. Product 3, instead, displayed approximately 50% fluorescence quenching upon the same condition in tetrahydrofuran. These results showed that we can design switchable fluorescent molecules that respond to mild inputs, like protonation by weak acid molecules, so that they can be used in biomedical conditions.

Notably, these probes have been designed to include a chemical entity that can be easily cleaved or modified for further functionalization with biomolecules, polymers or drugs. The aim of this work was to contribute to the development of a versatile platform of fluorescent molecules with potential applications as monitoring tools in drug delivery and biosensing.

## Supplementary Material

Probes characterisation

## Data accessibility

This article does not contain any additional data.
