# Supplementary material for: Synthesis of 1,8-naphthalimide-based probes with fluorescent switch triggered by flufenamic acid
Source: R Soc Open Sci. 2018 Jun 20;5(6):172137. doi: 10.1098/rsos.172137 (PMC6030310; doi:10.1098/rsos.172137)
Supplement: Probes characterisation [file rsos172137supp1.pdf]

Flufenamic acid as a trigger of 1,8-naphthalimide based fluorescent switches

G. Saito[1], D. Velluto[1,2] and M. Resmini[1]*

[1]Department of Chemistry and Biochemistry, SBCS, Queen Mary University of London, E1 4NS, London, UK

[2]Diabetes Research Institute and Cell Transplant Center – University of Miami Leonard M. Miller School of Medicine, Miami, FL 33136, USA

SUPPORTING INFORMATION

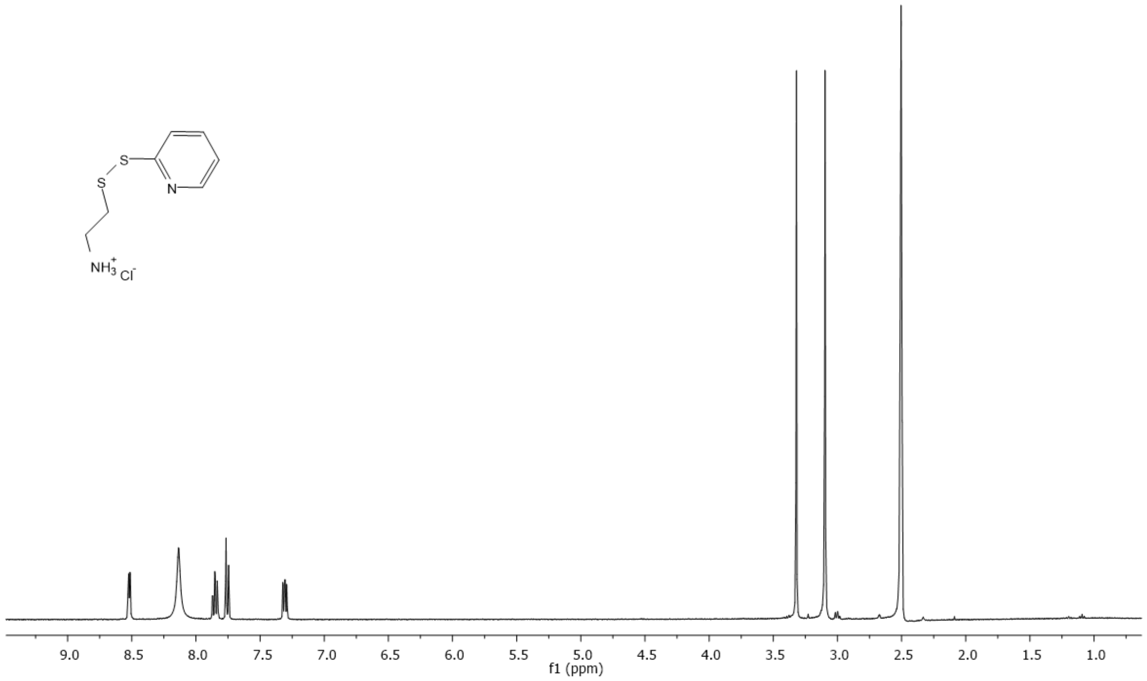

Figure S1. ^{1}H-NMR of product 5 in DMSO-$_{d6}$.

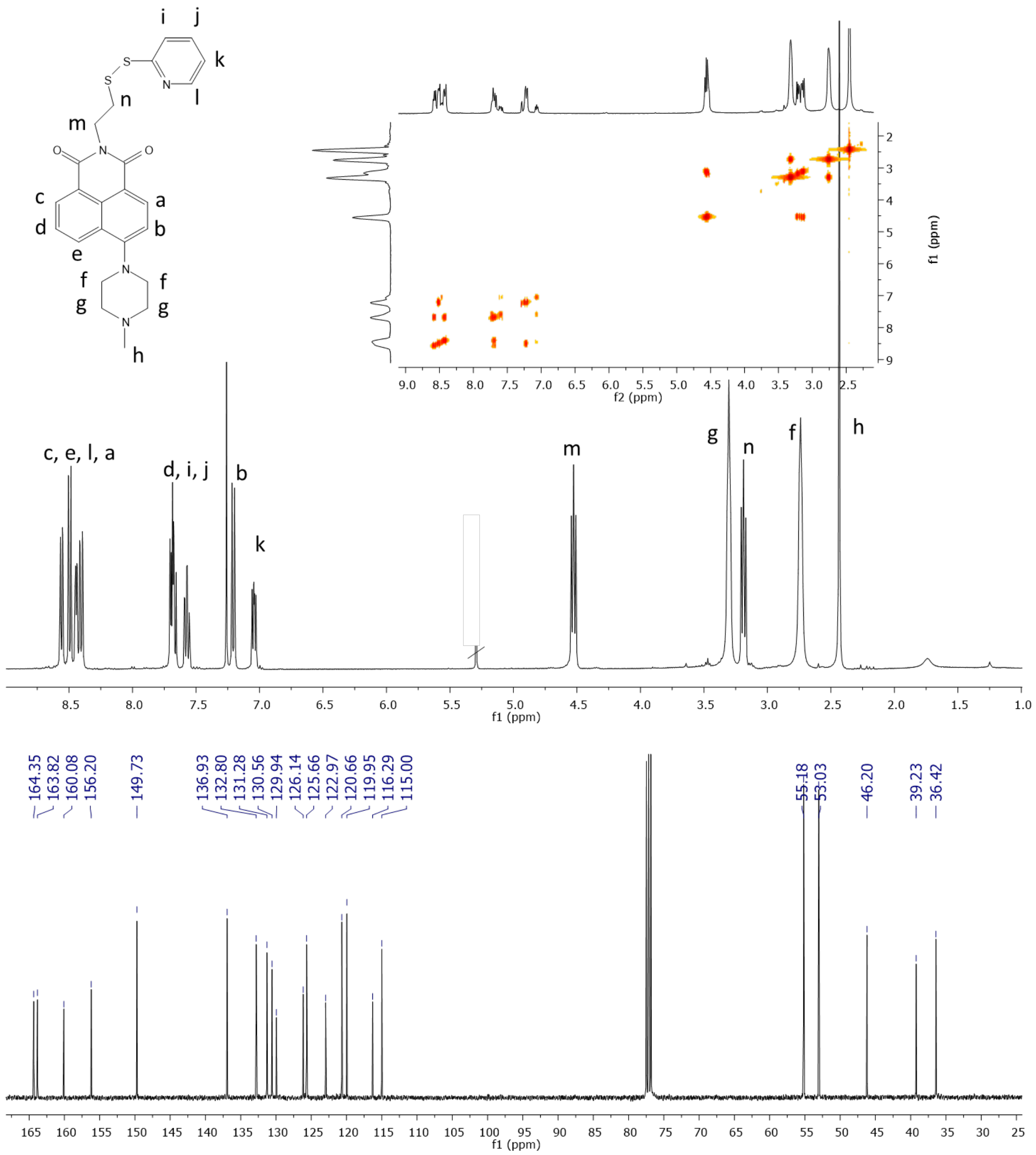

Figure S2. ^{1}H-NMR, COSY and ^{13}C-NMR of probe 1 in $CDCl_3$.

Probe 1 - Product B (figure S3): 6-(4-methylpiperazin-1-yl)-2-(2-yldisulfanyl)ethyl)-1H-benzo[de]isoquinoline-1,3(2H)-dione: ^{1}H–NMR (400 MHz, $CDCl_3$): δ 8.53 (1H, dd, J= 0.8 , 7.2), 8.47 (1H, d, J= 8), 8.38 (1H, dd, J= 0.8 , 8.4), 7.65 (1H, dd, J= 7.2 , 8.0), 7.18 (1H, d, J= 8), 4.53 (2H, t, J= 7.2), 7.28 (4H, s), 3.11 (2H, t, J= 7.2), 2.73 (4H, s), 2.43 (3H, s).^{13}C NMR (100 MHz, $CDCl_3$) δ 164.46 (s), 163.94 (s), 156.16 (s), 132.81 (s), 131.32 (s), 130.51 (s), 130.06 (s), 126.26 (s), 125.71 (s), 123.21 (s), 116.61 (s), 115.07 (s), 55.28 (s, 2C), 53.11 (s, 2C), 46.27 (s), 39.62 (s), 35.85 (s). FT-IR: cm^{-1} 2792.8 (-C-H Al), 1651.5 (-N-C=O), 1590.2 (-C=C- Ar), 1141 (C-N), 707 (-CH2-S). Melting point range 231-233 °C.

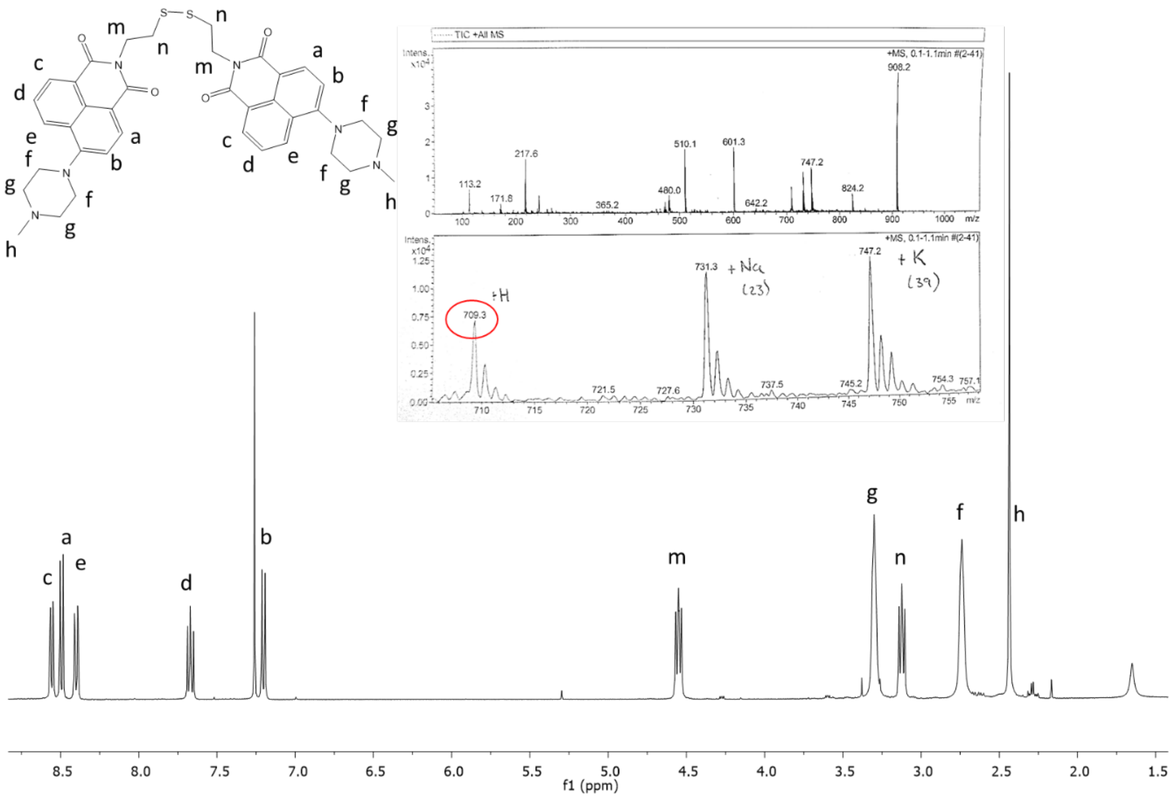

Figure S3. ^{1}H-NMR of dimer of probe 1 in $CDCl_3$ and LC-MS spectrum, showing the peak of the dimer at 708 M/z.

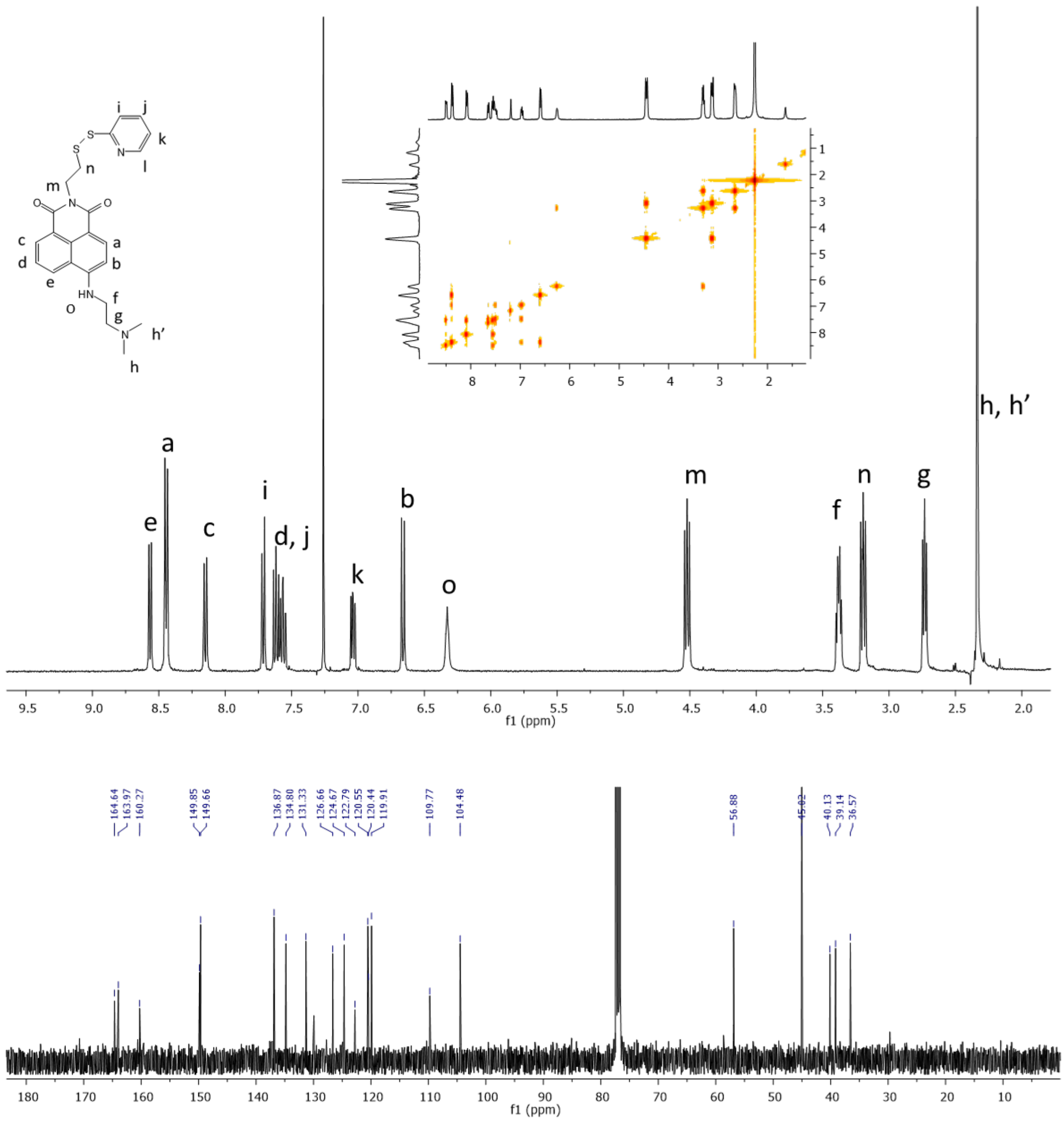

Figure S4. ^{1}H-NMR, COSY and ^{13}C-NMR of probe 2 in $CDCl_3$.

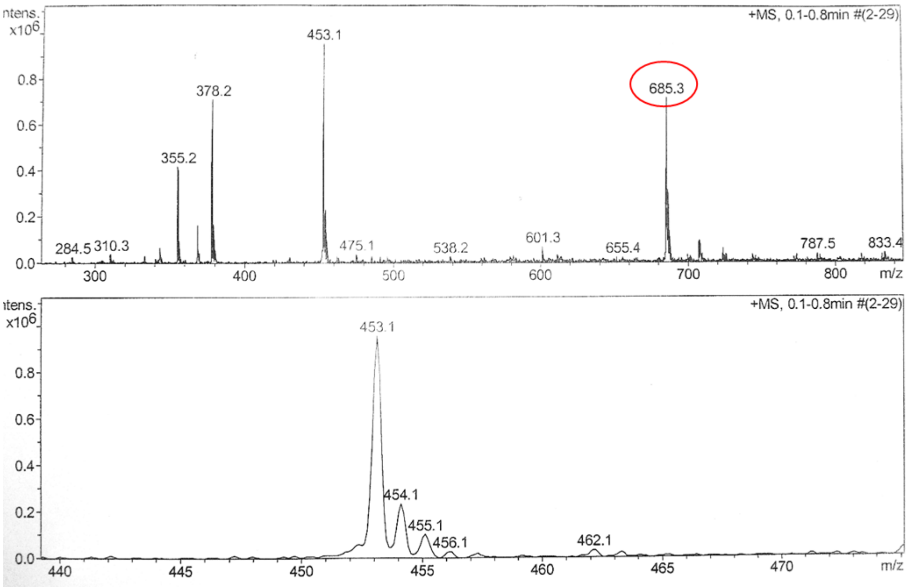

Figure S5. LC-MS of dimer of probe 2.

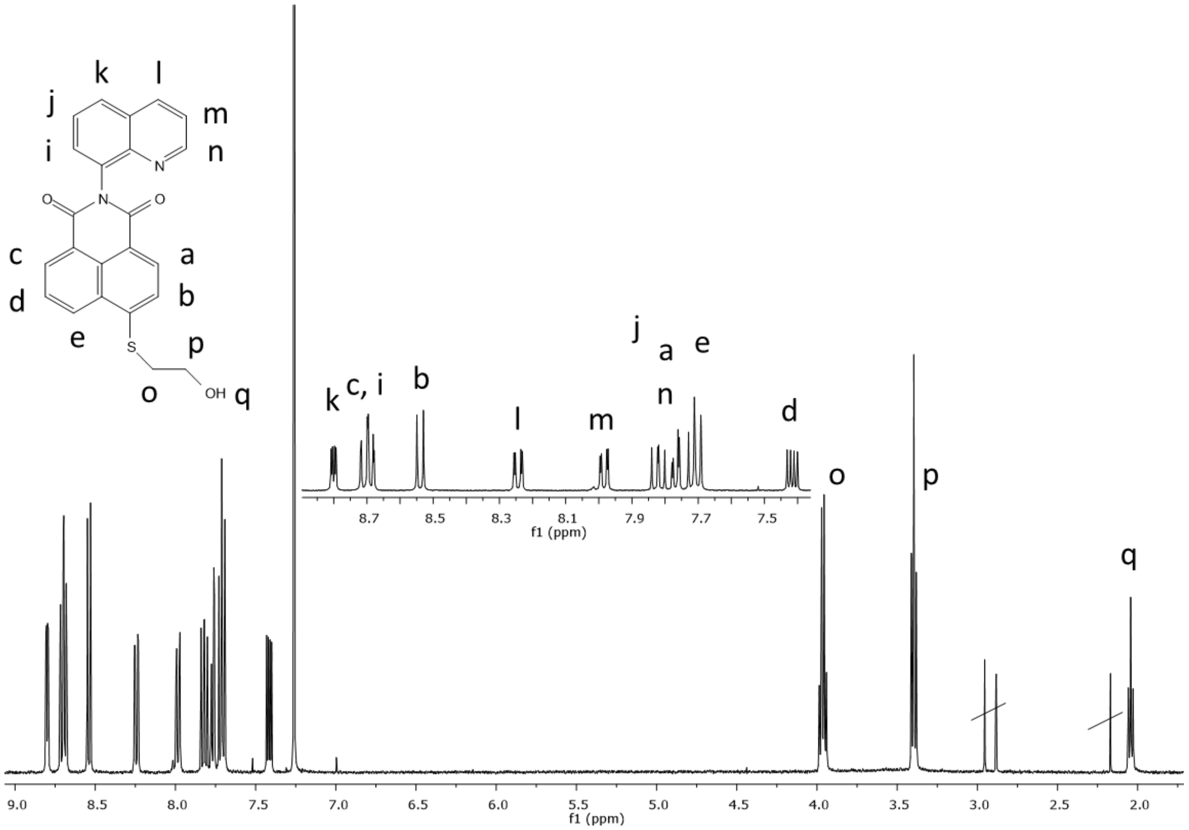

Figure S6. ^{1}H-NMR of probe 3 in $CDCl_3$.

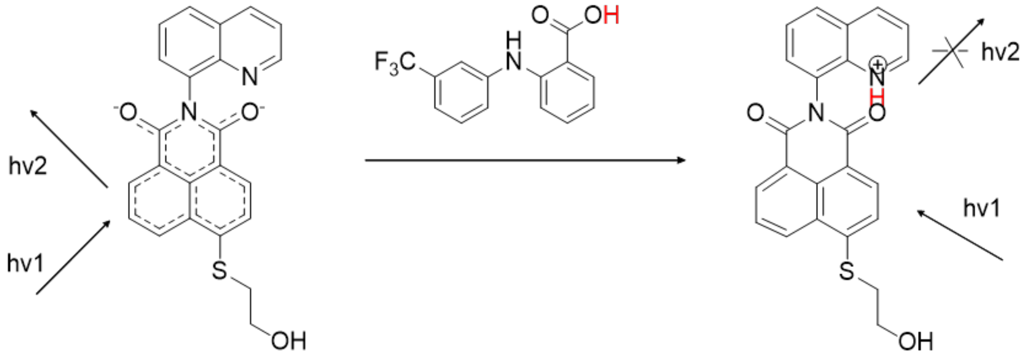

Figure S7. Proposed mechanism for fluorescence quenching (probe 3)

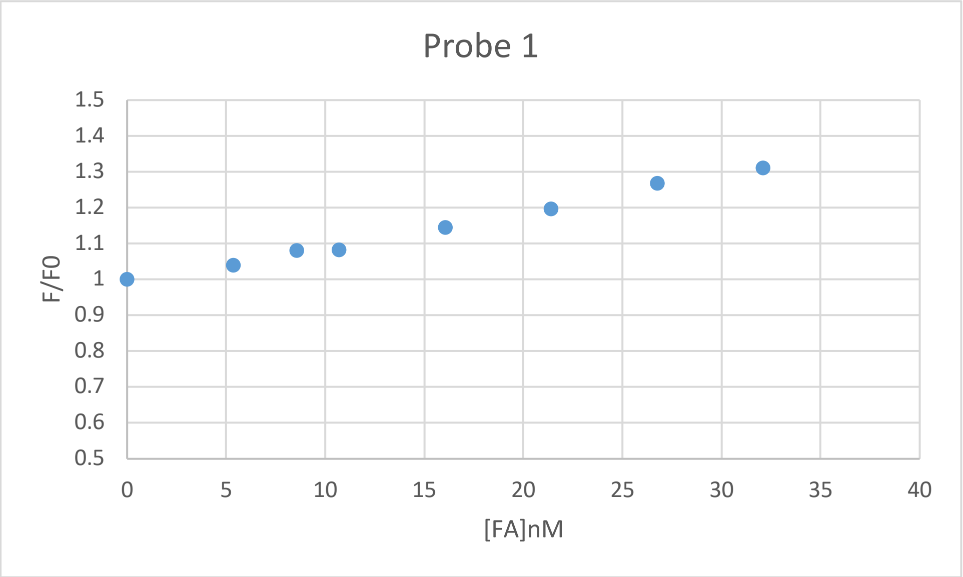

Figure S8. Probe 1: plot of F/F0 vs [FA] that show a linear correlation and no evidence of saturation
